# Chicago Medical Society

**Published:** 1867-07

**Authors:** 


### Proceedings of Societies.

### CHICAGO MEDICAL SOCIETY.

### FIBROID POLYPUS OF THE UTERUS.

Dr. Powell presented an interesting specimen of uterine tumor, which he had removed from a patient, 44 years of age. The patient had been married 22 years, had borne one living child, and experienced one miscarriage. She had uniformly enjoyed good health, except during the past five years. The first unpleasant symptom was a profuse menorrhagia, with bearing down pains, which usually lasted ten or twelve days. These symptoms continued two or three years, when the hemorrhage became so profuse and constant that the patient was compelled to remain in a recumbent posture. During the past two years, the anæmia and consequent nervous excitability have been alarming symptoms. The mucous membranes of the mouth and vagina were completely blanched, the discharge from the latter resembling thin mucilage.

On examination with the finger, the uterus was found high in the pelvis, the os being small but dilatable. The introduction of the finger into the cavity of the uterus, revealed a large tumor, attached by a short, thick pedicel near the fundus. The os was still further dilated by the fingers, when the tumor was seized by a pair of forceps and gently drawn down. The chain of the ecraseur was thus readily passed over the polypus to the pedicel, which was divided, with the loss of scarcely a drop of blood. The tumor, as may be seen, is of a firm, fibrous structure, spheroidal in shape, its long diameter being about two inches, and its short diameter about one and a-half inches in length. Its surface was covered by a thin, vascular membrane, which was readily separated from the solid structure beneath.

### CANCEROUS DISEASE OF THE STOMACH AND LIVER.

Dr. Fitch exhibited the stomach and liver of a female patient, 36 years of age, to whom he had been called the day before her death, but whose history he was unable to obtain.

The stomach, near the pyloric orifice, was much thickened and indurated by cancerous structure, the mucous membrane being much ulcerated. There was a large mass of cancerous growth in the liver, which was firmly attached to the stomach at the point where these organs normally lie in contact. There were, also, numerous nodules scattered over the lower surface of the liver, and also in its substance. The peritoneum was slightly affected.

### GLAUCOMATOUS EYE.

Dr. Holmes exhibited an eye, which he had recently removed from a female patient, 65 years of age, who had suffered for two years from frequent attacks of most agonizing pain in and around the orbit. The primary attack occurred in a distant town, two years ago. The description of the symptoms, as given by the patient herself, in connection with the appearance of the eye at the time of its removal, leave no doubt that the disease was acute glaucoma. Iridectomy had not been performed. During these two years, the patient was subject to frequent attacks of most terrible pain.

On examination, previous to the operation, the globe was found to be exceedingly hard, the cornea insensible to the touch, the pupil much dilated, the iris discolored and apparently atrophied, the lens opaque, and the conjunctiva slightly congested.

A free incision into the globe, as recommended by Hancock, was made, with the hope that the reduction of the great tension which existed would afford relief. The terrible pain, however, somewhat modified by the puncture, returned in a couple of days. Extirpation was deemed the most certain means of removing the distressing pain.

On dividing the globe, after the removal, the retina was found detached from the choroid and floating in a serous fluid, in form not unlike an umbrella nearly closed, a position in the central portions of the eye which this membrane must almost necessarily assume, when large quantities of fluid collect between it and the choroid.

### LARGE CONCRETION, NEARLY FILLING THE GLOBE.

Dr. Holmes also exhibited a specimen, with the following history:—A blind Scotchman, 48 years of age, entered the Chicago Charitable Eye and Ear Infirmary, with most violent pain in the right eye. He stated that at the age of 15 years, his right eye had been accidentally punctured by a thorn. The wound healed slowly, leaving the eye, however, for sixteen years, subject to frequent attacks of intraocular inflammation, attended with much pain. At the end of this long period, the other eye began to be somewhat painful and red; the pupil gradually contracted and finally closed; vision slowly diminished, as the disease progressed, till the patient was totally blind.

The patient's description of the progress of the disease in each eye is so intelligent and minute, that there can scarcely be a doubt that we have, here, an example in which a patient was permitted, by inexperienced physicians, not only to endure most terrible periodic pain for sixteen years, but also to suffer total loss of sight in the other eye by sympathetic inflammation. Early extirpation of the injured eye would almost certainly have relieved the patient of pain and preserved the other eye.

After total blindness had occurred, the poor patient was permitted to suffer, from time to time, the same agonizing periodic pain for *seventeen* years longer.

On entering the Infirmary, the left eye was atrophied, but not painful. The right eye, which had received the thorn, was in the following condition:—The conjunctiva was very œdematous, the atrophied cornea being almost totally concealed from view; there was not only great tenderness on pressing upon the lids, but excruciating pain in and around the eye.

On extirpating the globe, it was found atrophied and deeply grooved by the recti muscles, and very hard; the sclerotic was much thickened; the whole interior of the eye was filled with a hard, calcareous concretion; no trace of the iris or choroid could be found; there was but a remnant of the lens; the anterior part of the concretion was very thin—through this portion a needle could be easily passed into a small central cavity, which undoubtedly contains remnants of the retina and vitreous humor. The injury, probably, caused hyperplastic inflammation of the choroid. The products of this inflammation were deposited, from time to time, in layers, between the choroid and retina, the latter of which was forced into the central portions of the globe; finally, the softer portions of the exudation became absorbed, the calcareous material being left in the form of the solid concretion observed in the specimen, very hard, and, on being struck with metal, giving the resonance of marble.

